# Targeting the Epigenetic–Immune Axes in Hematologic Malignancies

**DOI:** 10.3390/cancers18182985

**Published:** 2026-09-15

**Authors:** Ravina Pandita, Evan F. Lind

**Affiliations:** 1 Graduate Program in Biomedical Sciences, Oregon Health & Science University, Portland, OR 97239 , USA; 2 Department of Molecular Microbiology and Immunology, Oregon Health & Science University, Portland, OR 97239, USA; 3 Knight Cancer Institute, Oregon Health & Science University, Portland, OR 97239, USA; 4 Department of Cell, Development and Cancer Biology, Oregon Health & Science University, Portland, OR 97239, USA; 5 Department of Pediatrics, Oregon Health & Science University, Portland, OR 97239, USA

**Keywords:** hematologic malignancy, immune suppression, epigenetic therapy, immune therapy

## Abstract

Despite significant advances in treatment for several hematologic malignancies, treatment resistance and relapse remain major challenges. Immune escape is recognized as a key mechanism that can lead to tumor evasion and treatment resistance in blood cancers. Among the various drivers of immune evasion, epigenetic dysregulation is a recurrent and central feature in these malignancies. Although epigenetic targeting therapies have shown benefit in blood cancers, relapse still occurs, indicating the need for improved therapies. This review focuses on epigenetic mechanisms causing immune evasion in blood cancers and discusses emerging therapies that focus on combining epigenetic modulators with immunotherapies to improve anti-tumor immunity. We focus on how epigenetic mechanisms, including DNA methylation, histone modification and chromatin remodeling, influence tumor cells or immune cell subsets including T cells, NK cells and macrophages to promote tumor growth. We then discuss immunomodulatory effects of epigenetic therapies including DNA methyltransferase inhibitors and histone deacetylase inhibitors and how they can restore immune cell function and limit tumor growth and progression. Finally, we highlight the potential of combining epigenetics with different immunotherapies including adoptive cell therapy and immune checkpoint blockade, while discussing their current limitations and suggesting ways to overcome this therapy resistance. A deeper understanding of the immune–epigenetic axis in blood cancers may facilitate the development of effective therapies that will benefit patients with hematologic malignancies.

## Simple Summary

Epigenetic therapies have shown clinical benefit across several hematologic malignancies, yet treatment resistance occurs. Immune evasion is increasingly being recognized as a major mechanism of treatment resistance. In this review we examine how modulating the immune system through epigenetic mechanisms may improve therapeutic outcomes in blood cancers including lymphoma, leukemia and multiple myeloma. We highlight how epigenetic alterations can promote evasion from immune cell surveillance by impairing several immune cell populations including T cells, natural killer cells and macrophages. We then explore strategies to improve anti-tumor immunity using epigenetic therapies that target DNA methylation and histone modifications. Lastly, we consider the potential of combining epigenetic agents with immune-targeting therapies as a promising approach in these malignancies.

## 1. Introduction

### 1.1. Hematologic Malignancies

Hematologic malignancies comprise approximately 200,000 cases annually and 57,000 deaths in the United States every year [1]. They are mainly characterized by abnormalities during hematopoiesis and differentiation. They can be broadly characterized into lymphoid and myeloid malignancies depending on both their cellular origin and final differentiation phenotype.

Leukemias are a group of hematologic malignancies that are characterized by abnormal clonal proliferation of hematopoietic cells in the bone marrow, blood and other tissues, leading to uncontrolled proliferation and impaired differentiation. They are caused by genetic and epigenetic modifications impacting hematopoietic cells at different stages of differentiation and can be classified into myeloid or lymphoid types based on their origin. They are heterogenous and mainly characterized into acute or chronic forms. Acute leukemia, mainly acute myeloid leukemia (AML), is one of the most aggressive blood cancers, with a poor survival rate [2]. Because of their heterogeneity, treatment strategies are diverse but commonly include chemotherapy, targeted therapies and hematopoietic stem cell transplants.

Lymphomas represent a heterogenous type of hematologic malignancy. Lymphoma is the seventh most common cancer in the US [3]. This cancer results from clonal proliferation of lymphoid cells, most commonly B cells but also T cells and natural killer (NK) cells. There are multiple types of lymphomas that we can identify, but they are classified into two main categories: Hodgkin lymphoma (HL) and non-Hodgkin lymphoma (NHL). These cancers also arise from a variety of genetic and epigenetic aberrations, contributing to variable outcomes clinically. The most common types of treatment comprise cytotoxic chemotherapy and targeted therapies.

Multiple myeloma (MM) is a blood cancer that comprises plasma cells, which are terminally differentiated B cells residing in the bone marrow. While its exact cause is unknown, plasma cell disorders such as Monoclonal Gammopathy of Undetermined Significance (MGUS) can put one at a higher risk [4]. Several therapies are used in MM including chemotherapy, targeted therapies including proteasome inhibitors and stem cell transplant, but therapy resistance occurs due to several mechanisms including genetic and epigenetic alterations [5,6,7,8].

### 1.2. Immune Evasion in Hematologic Malignancies

**Figure 1:**
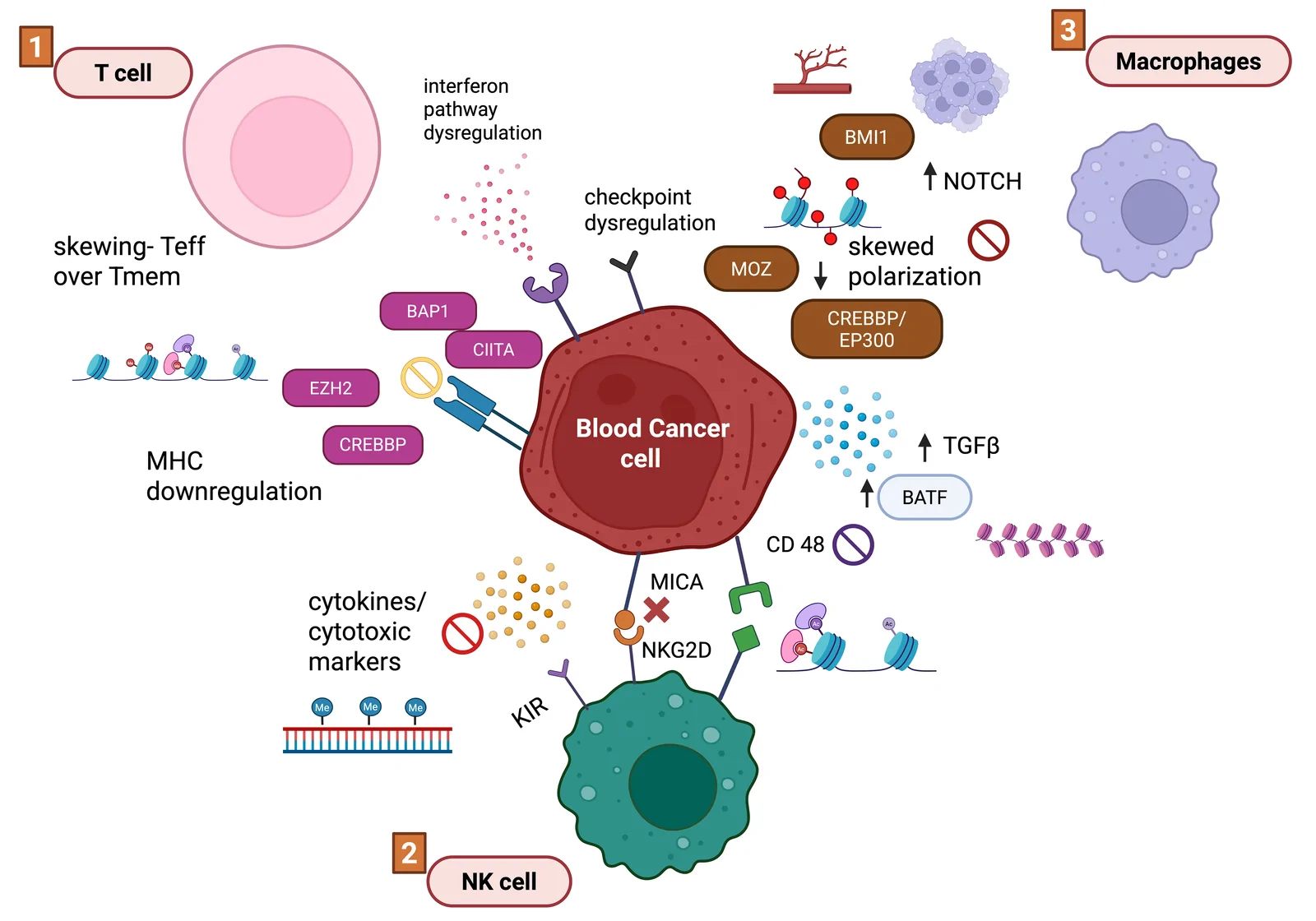
Epigenetic mechanisms of immune evasion in hematologic malignancies. Schematic illustrating how epigenetic alterations in both malignant and immune cells contribute to immune evasion in hematologic malignancies. (1) In malignant cells, dysregulation or mutation of epigenetic regulators (e.g., EZH2, CREBBP, CIITA, BAP1) reduces MHC class I and II expression, impairing T cell recognition [88,89,90,91]. Another mechanism involves skewing T cells towards a memory-like phenotype, altered checkpoint signaling, and suppression of cytokine production, collectively limiting anti-tumor immunity [92,93,94]. (2) In NK cells, epigenetic alterations, including histone modifications and DNA methylation, reduce the expression of activating NK cell receptors (e.g., KIR) or downregulate AML ligands (e.g., CD48, MICA), thereby impairing NK cell cytotoxicity [95,96,97]. These alterations can also promote BATF expression, suppress NK cell function and enhance the release of immunosuppressive factors, such as TGF-β, by AML cells [98]. (3) Epigenetic regulators, including CREBBP/EP300, MOZ, BMI1 and DNMT3A, dampen macrophage function by promoting a pro-tumorigenic phenotype [99,100,101,102]. This reprogramming alters macrophage proliferation and angiogenesis, ultimately supporting tumor progression through pathways including NOTCH signaling. Figure created in bioRender.com.

Evasion of the immune system is a hallmark of cancers including hematologic malignancies (Figure 1) [9,10,11]. While immune evasion is widely studied in solid tumors, emerging evidence suggests its significance in blood cancers. In AML, several studies have shown profound impairment of T cells in blood and bone marrow of patients both at diagnosis and relapse post-treatment [12,13,14]. The abundance and functional status of T cells in patients are predictive of both overall survival and relapse after chemotherapy. These associations show that T cells may play a critical role in disease modulation in AML. Patients with AML also have an increased frequency of regulatory T cells (Tregs) compared to healthy controls, likely impairing T cell function [15,16,17]. The antigen specificity of these Tregs is currently not known and is a topic of current research. It is also well known that myeloid-derived suppressor cells (MDSCs), inhibitory checkpoint markers (such as PD-L1, CTLA-4, VISTA), secretion of soluble immunosuppressive mediators, and many other factors also contribute to immune evasion in AML [11,18,19,20].

Similar mechanisms of immune escape are observed in lymphomas as well, where malignant cells reduce antigen presentation capabilities, downregulate expression of co-stimulatory molecules, and produce immunosuppressive cytokines and tumor-suppressive cells such as Tregs and tumor-associated macrophages (TAMs) to escape anti-tumor immunity [9,21,22]. In Hodgkin’s lymphoma, up to 97% of cases involve upregulation of PD-L1 and PD-L2 through various genetic means [23,24], suggesting immune evasion via checkpoint expression.

In multiple myeloma, emerging evidence shows that immune evasion is driven in part by immune editing by genetic alterations and expansion of tumor subclones [25]. Similar mechanisms of immune escape involving antigen escape and tumor-mediated immune suppression [26] are also observed in this malignancy. There is an additional modulation of the immune system by gut microbiota where several metabolites and short-chain fatty acids may regulate immune responses [27].

### 1.3. Immune-Targeting Therapies in Blood Cancers

Significant efforts are focused on developing immunotherapies for these cancers. In fact, immune-based strategies for blood cancers date back to the 1960s, when the first allogeneic stem cell transplant was performed [28]. Donor immune cells including T cells post-transplant can cause the graft vs. leukemia effect, where donor immune cells recognize and attack remaining tumor cells [29,30]. Donor lymphocyte infusion after allogeneic transplant is a way to induce this anti-tumor effect, showing potential for adoptive immune therapy in leukemias [31]. The first FDA-approved immunotherapy for hematologic malignancies is rituximab [32], a monoclonal antibody which targets CD20 and is widely used for several B cell malignancies and lymphomas. This was recently followed by more successful therapies against CD19 and CD20 for relapsed/refractory B cell malignancies [33,34].

Another promising class of immunotherapy that has emerged is bispecific antibodies (BsAbs). For example, a CD3 x CD19 bispecific T cell engager, blinatumomab, is FDA-approved for relapsed/refractory precursor B cell acute lymphoblastic leukemia (pre-B-ALL) [35]. Multiple BsAbs targeting BCMA x CD3 are also approved for patients with relapse or refractory multiple myeloma [36]. Several other bispecific antibody targets such as CD33, FLT-3 and CD123 are being evaluated for their clinical potency in leukemias [37].

Chimeric antigen receptor (CAR-T) cell therapies have changed the therapeutic landscape for certain hematologic cancers [38,39,40]. CAR-T cell therapies such as Kymriah and Yescarta target CD-19 and are approved for certain B cell lymphomas and leukemias. Another CAR-T cell product approved for multiple myeloma is CARVYKTI, which is a BCMA-targeting CAR-T cell, with more novel targets being an active area of investigation [41,42].

Due to the success of checkpoint blockade in solid tumors and evidence that several hematologic malignancies use inhibitory checkpoint pathways to escape immune recognition, these pathways represent a promising target for therapy. PD-1 blockade is approved in Hodgkin’s lymphoma [43] and several clinical trials are currently investigating additional immune checkpoints including CTLA-4, LAG-3, and TIGIT, with some showing encouraging results [44,45].

Despite the therapeutic advances, multiple clinical trials have demonstrated limited efficacy for immune therapies. This is likely due to multiple factors including emergence of resistance due to antigen loss or escape, secretion of immune-suppressive factors, treatment-related toxicity, immune checkpoint activation and T cell exhaustion in blood cancers. This underscores the need to elucidate mechanisms of immune evasion in hematologic malignancies to develop effective therapeutic responses.

### 1.4. Epigenetic Dysregulation in Blood Cancers

Multiple biological factors may contribute to a lack or limited efficacy of immunotherapy in hematologic cancers, with epigenetic dysregulation being a key contributor. Epigenetic modifications including DNA methylation, histone modifications and chromatin remodeling are recognized as drivers of oncogenes, disease progression, therapy resistance and immune dysfunction [46]. In a subset of patients with AML, mutations in epigenetic regulators such as TET2, DNMT3A and IDH1/2 lead to abnormal gene expression and are frequently associated with poor prognosis [47,48,49]. In fact, DNMT3A mutations can cause leukemia progression by activation of several tumor-permissive transcriptional pathways [50,51,52]. Consequently, inhibitors targeting the DNA-methylation-regulating genes have been developed and are being investigated and used as treatment strategies in hematologic malignancies.

DNA methylation is a critical epigenetic mechanism involving the addition of methyl groups to the 5′ position of cytosine in DNA. This modification is an important mechanism for regulating cellular processes including control of gene expression in physiological conditions [53]. However, in cancer, as mentioned, DNA methylation is often aberrantly regulated, which can activate oncogenes. Emerging evidence suggest that abnormal methylation may contribute to immune escape [54,55]. Importantly, these aberrant methylation signatures can be used as a prognostic biomarker in several cancers [56].

Azacitidine and decitabine were first developed as cytotoxic with the intent of blocking replication. The mechanism of action of these cytidine analogs was later found to inhibit DNA methylation [57]. They work by inhibiting DNA methyltransferase and promoting DNA hypomethylation, which can restore the expression of silenced tumor suppressor genes including p15, p16 and MLH1 [58,59,60]. Due to their therapeutic efficacy, azacytidine and decitabine are used to treat many hematologic malignancies including MDS, AML and CMML [61,62,63]. Azacytidine and decitabine are commonly used as a front-line therapy for patients unfit for intensive chemotherapy and can induce favorable clinical responses [46,64].

In addition to DNA methylation, histone modifications are also important in hematologic malignancies. Histone modifications regulate gene expression by influencing chromatin structure and accessibility. Histone acetylation, mediated by histone acetylases (HATs), catalyzes the addition of acetyl groups to the histone tails, resulting in a transcriptionally active chromatin state called euchromatin [65]. Conversely, removing these acetyl groups by histone deacetylases (HDACs) promotes closed chromatin conformation into a transcriptionally repressed state called heterochromatin [66].

Mutations targeting histone acetylation such as CBP and p300 are present in subsets of leukemia and lymphomas and are associated with adverse clinical outcomes [67,68,69]. Targeting with histone deacetylase inhibitors (HDACis) can reverse these abnormal histone modifications and has shown clinical success in cutaneous T cell lymphoma, peripheral T cell lymphoma and multiple myeloma [70,71].

Another important epigenetic regulator is EZH2, a histone methyltransferase. Mutations in EZH2 are frequently observed in leukemias and lymphomas [72,73]. These mutations contribute to disease progression and are associated with poor clinical outcomes in patients. As a result, therapies targeting EZH2 mutations have also gained critical interest and continue to be investigated [74,75].

Given the importance of targeting the epigenome, considerable efforts have been directed towards developing these therapies alone or in combination with other agents. As mentioned, some of these epigenetic therapies including hypomethylating agents, IDH inhibitors, and HDACis are already in use in clinics for hematologic malignancies such as MDS, AML, and multiple myelomas [76,77,78].

### 1.5. Impact of Epigenetic State on Immune System Evasion

Previous reviews have focused on tumor-intrinsic mechanisms and direct anti-tumor effects of epigenetic modulation and therapies. Beyond tumor-intrinsic effects, extensive evidence demonstrates that epigenetic remodeling is central for immune modulation in blood cancers, contributing to both immune activation and evasion. This highlights a critical intersection between immune escape and epigenetic dysregulation, suggesting targeting the epigenetic–immune axis has the potential to develop novel impactful therapies.

This review focuses on this interplay between epigenetics and the immune system in hematologic malignancies, with an emphasis on how epigenetic mechanisms drive immune evasion, and explores emerging epigenetic targeting strategies that can restore anti-tumor immune responses.

Epigenetic mechanisms can regulate T cell dysfunction via several mechanisms involving T cell-intrinsic changes impacting their differentiation, exhaustion and checkpoint expression, as well as tumor-intrinsic mechanisms that impair T cell recognition and activation. These are discussed below.

## 2. T Cell-Intrinsic Epigenetic Regulation of Dysfunction and Exhaustion

### 2.1. T Cell Differentiation and Exhaustion

CD8+ and CD4+ T cells play a central role in mediating anti-tumor immunity by recognizing tumor antigens presented by major histocompatibility complex (MHC) class I and class II respectively. They promote tumor killing by secreting cytotoxic molecules like perforin and granzyme as well as cytokines that enhance tumor killing and increase tumor sensitivity to immune attack [79,80].

All stages of T cell development and function, including activation, differentiation, and memory development, are regulated by epigenetics [81]. T cell exhaustion is a dysfunctional T cell state induced by persistent antigen stimulation as seen in chronic viral infections and cancer, and is characterized by a lack of proliferative and functional abilities [82]. Terminally exhausted T cells have a different chromatin landscape than other T cell subsets including effector and memory T cells [83,84,85], highlighting that epigenetic remodeling can enforce this dysfunctional state. In fact, the genes encoding key transcription factors of T cell exhaustion including TOX, TCF-1 and BATF undergo chromatin modifications and DNA methylation changes to maintain different stages of T cell exhaustion [86,87].

In a spontaneous murine model of chronic lymphocytic leukemia (CLL), epigenetic mechanisms may drive antigen-specific T cells to short-lived effectors over memory cells. This skewed differentiation was associated with a reduced chromatin accessibility at memory-related gene loci and represses the transcriptional machinery required for formation of memory cells [92]. Numerous studies have shown that memory cells persist longer, exhibit superior proliferative capabilities, and are essential for tumor control compared to other T cell subsets. In patients, the presence of memory populations correlates with a better prognosis across several cancers [103]. Even in the context of exhaustion, the presence of a stem-like state, with memory-like characteristics, is favored as these cells can respond to checkpoint blockade [104]. Zebley et al. profiled CD19 CAR-T cells after infusion in patients with acute lymphoblastic leukemia and they found that after infusion, these T cells lose expression of stem-like memory markers including TCF7 and LEF1 over time [105]. This was accompanied by a DNA methylation reprogramming where exhaustion-related genes such as TOX and BATF were demethylated, suggesting a shift towards exhaustion.

Another study that focused on gene expression analysis of CD8+ T cells in bone marrow of patients with AML found that genes related to T cell activation and differentiation including NF-κB, NOTCH, WNT and TCR signaling were downregulated. Further analysis found that histone deacetylation mediated the gene repression of these immune genes in patients [93]. Collectively, these are examples of how epigenetic dysregulation is associated with several aspects of T cell biology in leukemias including T cell function, activation, differentiation, and memory formation.

### 2.2. Checkpoint Pathways in T Cells

Immune checkpoint inhibitors are revolutionizing immunotherapy responses in several cancers [106,107,108,109]. One major mechanism by which tumor cells escape immune surveillance is by upregulating inhibitory checkpoint ligands, leading to suppression of T cell function [82]. In the tumor microenvironment, modulating checkpoint receptor expression on T cells can also be a potential mechanism of immune escape [110]. Due to the success of checkpoint blockade in solid tumors, multiple clinical trials have been conducted in AML. Clinical trials have and continue to evaluate the efficacy of PD-1 blockade with nivolumab and pembrolizumab and targeting CTLA-4 using ipilimumab, alone or in combination with other immune therapies in AML. Clinical responses to date have been varied. For example, one study showed that nivolumab alone as a single agent in high-risk AML only benefitted a small subset of patients, and was not successful in elimination of minimal residual disease, which can contribute to relapse [111]. Another study showed that after chemotherapy, treatment with pembrolizumab resulted in a modest survival benefit but did not induce remission in most patients as a monotherapy [112]. Therefore, combinations of checkpoint blockade with other therapeutics are actively being explored [113,114].

Epigenetic mechanisms are increasingly associated with regulation of checkpoint expression. In bone marrow samples from patients with AML, immune checkpoint receptor genes including BTLA, CD244, CD96, etc., were downregulated in both CD4+ and CD8+ T cells, and these genes were found in regions of histone remodeling. Mechanistically, this suppression may be regulated by PAC1, a phosphatase with histone deacetylation activity, suggesting epigenetic control of checkpoint-related transcriptional programs may modulate T cell dysfunction [94].

In contrast, CD8+ T cells derived from peripheral blood of patient samples with CLL show elevated PD-1 expression which is associated with hypomethylation in the enhancer region of PDCD1 gene [115]. Genome-wide DNA methylation analysis also revealed that several other genes significant for T cell function such as TCRA, NFATC1, and HLA class II genes are epigenetically regulated in the T cells of patients with CLL [115]. Similarly, in AML and MDS, studies have reported that PD-1 expression is associated with methylation of its promoter [116,117]. These studies highlight context-dependent epigenetic regulation of checkpoint expression across hematologic malignancies. This also suggests that epigenetic modification of checkpoint markers can contribute to altered T cell states and may change responsiveness to checkpoint blockade, although this relationship requires further investigation.

## 3. Tumor-Intrinsic Mechanisms of T Cell Evasion

### Antigen Presentation and Immune Recognition

Regulation of antigen presentation to activate T cells that can recognize and kill tumors is an important component of anti-tumor immunity. Loss or downregulation of MHC class I and II is a hallmark mechanism of immune escape in many hematologic malignancies [9,19,25], especially after stem cell transplants, contributing to disease relapse. In fact, HLA loss is uncommon at diagnosis but is more frequently observed at relapse and this weakens the graft vs. leukemia (GVL) effect, which is critical for efficacy of transplantation in terms of relapse-free survival [118,119,120]. In fact, a recent study performed targeted sequencing on 25 patients using paired samples from before and after transplant as well as after relapse. They found loss of HLA class I as a major contributor of relapse in these patients [119]. Another study evaluating 34 patients with AML that had undergone relapse after transplant found that downregulation of MHC class II genes was observed in half of the patients and suggested that this suppression was mediated by epigenetic mechanisms [121].

In fact, many studies show that epigenetic factors play a critical role in the process of antigen loss through MHC repression. Mutations in DNMT3A are associated with impaired antigen presentation in AML [122], while another study demonstrated that relapse was associated with a loss of MHC class II expression in this disease. Using paired patient samples at diagnosis and relapse, and patient derived xenograft models, it was shown that this loss was driven by a decrease in chromatin accessibility which is regulated by polycomb repressive complex 2 (PRC2) [123]. PRC2 works as a chromatin repressor, and is a part of the EZH2 complex, which is a key histone methyltransferase [124]. Notably, PRC2 dysregulation is a recurring theme in both solid and hematologic cancers [125]. This study also highlighted that not only CD8+ T cells but also CD4+ T cells play an essential role in the GVL effect, underscoring the importance of MHC II class loss [123]. Another study in AML using patient data showed that aberrant EZH2 activity can downregulate MHC class I and II expression [88]. Similarly, mutations in EZH2 are associated with a decreased lymphocyte infiltration as well as impaired antigen presentation through MHC class I and class II molecules [126] in diffuse large B cell lymphoma (DLBCL) as well, reinforcing how epigenetic repression can lead to immune evasion. Due to its epigenetic modifying properties, EZH2 is a promising target for reversing immune evasion, and will be discussed later in this review. Likewise, follicular lymphomas with mutations in the histone acetyltransferase CREBBP are accompanied by downregulation of MHC class II genes in patient samples and mouse models [89,127]. This loss of MHC II was correlated with a decreased infiltration of several T cell subsets in CREBBP-mutated tumors, suggesting a role for this chromatin modification in immune evasion in follicular lymphomas with this mutation. These examples highlight how epigenetic mechanisms may repress MHC expression across several hematologic malignancies.

Additionally, epigenetic mechanisms may also disrupt antigen presentation machinery. Mutations in class II major histocompatibility complex transactivator (CIITA), a key regulator of MHC class II expression, lead to aberrant antigen presentation [128,129,130,131]. Growing evidence suggests that CIITA is epigenetically modulated by DNA methylation and histone modifications [128,129,132,133]. A study across multiple hematologic malignancy cell lines and patient samples with AML found that CIITA expression was downregulated and correlated with reduced expression of HLA-DR [90]. DNA methylation was identified as a potential mechanism for this suppression. In B cell lymphoma mouse models and cell lines, loss of histone deubiquitinase BRCA1-associated protein 1 (BAP1) promotes increased tumor burden by downregulating MHC class II expression by suppressing multiple class II genes, including CIITA [91]. In the absence of BAP1, MHC class II genes adopt a repressed chromatin state which is associated with polycomb repressive complex 1 (PRC1) activity, sustaining decreased MHC II expression and promoting immune escape. A study in multiple myeloma using multiple experimental systems also found that the loss of histone demethylase KDM6A leads to a decrease in CIITA and NLRC5, another critical regulator of MHC expression. This decrease further suppressed antigen presentation and led to immune suppression in this malignancy [134].

Together, these mechanisms illustrate epigenetic regulation can influence immune escape at multiple levels—T cell-intrinsic regulation via differentiation, exhaustion, memory formation and checkpoint expression. Another layer is tumor-intrinsic epigenetic modifications via impaired antigen presentation and immune recognition. Therefore, understanding how these epigenetic mechanisms contribute to therapy resistance and immune escape is critical to optimize immune therapy strategies in these cancers.

## 4. Tumor Evasion of NK Cell Recognition and Function

Epigenetic mechanisms can also dysregulate NK cell responses by NK cell-intrinsic changes as well as tumor-intrinsic mechanisms that impair NK cell recognition. Both topics are discussed in this section.

### 4.1. NK Cell-Intrinsic Epigenetic Regulation of Dysfunction

Natural killer cells are important in mediating anti-tumor immunity in hematologic malignancies. Unlike T cells, NK cells do not require tumor antigen presentation via MHC molecules but recognize tumor cells by receptor–ligand interactions [135]. They eliminate tumor cells by releasing cytotoxic molecules like perforin and granzyme, as well as secreting inflammatory cytokines including TNF-α and interferon-γ [136,137].

Emerging evidence highlights the essential role of epigenetics and chromatin-associated transcription regulators in modulating NK cell dysfunction in multiple disease contexts including hematologic malignancies [138,139,140]. As mentioned earlier, mutations in TET2 are common in tumor cells of patients with MDS and AML [141]. A recent study showed that these mutations have functional outcomes regulating NK cells directly. TET2 mutations in NK cells lead to DNA hypermethylation of an NK cell receptor, killer immunoglobulin-like receptor (KIR), leading to a decrease in its expression [95]. These mutated cells also have reduced expression of cytotoxic molecules and cytokines required for tumor cell killing, supporting a role of DNA methylation in regulating NK cell cytotoxicity and function in hematologic malignancies.

Another epigenetic mechanism by which NK cells may be dysregulated in hematologic malignancies involves the transcription factor BATF, a chromatin-associated regulator that can modulate immune cell function [142,143]. BATF has established roles in several hematologic malignancies and contributes to the maturation and differentiation of leukemia cells [144,145]. A recent study demonstrated that BATF can impair NK cell responses in AML and MDS. In AML, the TGF-β/SMAD signaling pathway promotes dysfunctional NK cells, and inhibition of this pathway along with NK cell adoptive transfer restored NK function and achieved tumor control in leukemia mouse models [98]. Mechanistically, NK cells in these patients with AML had an upregulation of BATF and its targets. Further studies confirmed that this regulation was mediated by SMAD2/3 and TGF-β that was released by leukemia cells.

This is particularly significant as BATF is a critical transcription factor involved in T cell exhaustion [146,147]. Its role in NK cell dysfunction suggests a broader paradigm where epigenetic regulators of T cell exhaustion may also regulate NK cell states. These findings open the door for investigating additional T cell exhaustion markers in the context of NK cell dysfunction in hematologic malignancies. Future studies should aim to identify such shared and unique exhaustion pathways in both these cell types.

### 4.2. Tumor-Intrinsic Epigenetic Mechanisms of NK Cell Evasion

Similar to checkpoint pathways in T cells, tumor cells may evade NK cell surveillance through epigenetic modulation of checkpoint ligand–receptor interactions [148]. For example, NKG2A is an inhibitory receptor on NK cells which has ligands expressed on cancer cells, but these ligands can be dysregulated in hematologic cancers [96], limiting NK cell activity and cytotoxicity. Studies show that epigenetic repression may lead to this impairment. A notable example is CD48, a member of the SLAM family that functions as an activating ligand or receptor recognized by NK cells in AML [149,150]. AML cells evade NK cell surveillance by downregulating CD48 by hypermethylation and reduced acetylation of this gene [97,151], altering NK cell responses by impairing their killing capacity in vitro. Another study used AML cell lines and patient samples and found that the ligands for NKG2D, including MICA and UBLP1/2, are often downregulated and hypermethylated in AML, resulting in their decreased gene expression [152]. Consistent with these findings, epigenetic mechanisms regulating NKG2D ligands are observed in multiple myeloma [153].

These studies demonstrate that epigenetic alterations can contribute to NK cell dysfunction by multiple mechanisms including intrinsic NK cell dysfunction, modifying exhaustion-associated programs and tumor-regulated evasion of NK ligand–receptor interactions.

## 5. Epigenetic Regulation of Macrophage-Mediated Immune Evasion

Macrophages are central regulators of immune responses in tumors and exist along a spectrum of states, classically characterized as pro-inflammatory, immunostimulatory-like or anti-inflammatory, pro-tumorigenic state [154,155]. Tumor-associated macrophages (TAMs) mainly exhibit an immunosuppressive phenotype and contribute to immune evasion and are often associated with worse patient outcomes in cancers [156]. In hematologic malignancies, macrophages may promote immune escape by secreting immune-suppressive cytokines such as TGF-β and IL-10 [157]. Emerging evidence highlights that macrophages are influenced by epigenetic remodeling as well, which regulates their polarization, function and tumor promoting capacity.

Chromatin accessibility and histone modifications regulate macrophage differentiation and function. One epigenetic regulator involved in macrophage skewing is monocytic leukemia zinc-finger protein (MOZ), a histone acetyltransferase which is critical for normal hematopoiesis and stem cell maintenance [158]. MOZ acetylates H3 and H4 histones, therefore regulating chromatic accessibility and gene expression programs [159]. In AML, decreased expression of MOZ is related to poor clinical outcomes and contributes to leukemia progression [160]. Experimental knockdown of MOZ in tumor cells in culture increased tumor burden and suppressed pro-inflammatory macrophage activation [102]. Mechanistically, MOZ mediated histone acetylation, which regulates signaling pathways essential for macrophage polarization, underscoring how epigenetic remodeling can influence macrophage polarization and tumor outcomes in leukemia. Studies in other disease settings have also shown the role of MOZ family members in regulating macrophage activation and polarization programs [161,162].

DNMT3A dysregulation may also impact macrophage polarization and function [163,164]. Knocking out or inducing a loss-of-function mutation in DNMT3A using human AML cell lines or xenograft models caused macrophage dysfunction [99]. Upon suppressing DNMT3A, a decrease in the production of pro-inflammatory cytokines and a shift towards an immunosuppressive phenotype were observed. This was accompanied by a defect in killing capacity of these pro-inflammatory macrophages and an increased tumor burden. Similarly, in DLBCL, mutations in the histone acetyltransferase CREBBP or EP300 skew macrophage polarization towards the immunosuppressive phenotype by enhancing the NOTCH signaling pathway [100]. This alteration correlates with accelerated tumor growth. EP300 can also regulate macrophage polarization in other disease settings [165,166]. Similarly, studies in multiple myeloma have demonstrated that epigenetic mechanisms can influence macrophage polarization [167,168], further supporting the role for epigenetic regulation of macrophage phenotypes in blood cancers.

In several tumors, including multiple myeloma, breast and ovarian cancer, increased infiltration of TAMs is associated with poor prognosis and therapy resistance [169,170]. Accumulating evidence suggests that the function of these macrophages may also be regulated at the epigenetic level [171]. A histone modifier, B cell-specific moloney murine leukemia virus integration site 1 (BMI1), is a member of the polycomb-group of proteins that have multi-faceted roles in stem cell maintenance and cancer growth and also regulate macrophages [172,173]. BMI1 knockdown in multiple myeloma cells reduced macrophage proliferation and angiogenesis capacity and altered their chemoresistance property, which contributes to their pro-tumor activity [101]. Although the specific epigenetic mechanisms in this context were not fully elucidated, it is well known that BMI1 can monoubiquitinate histones leading to transcriptional gene repression [174]. Mechanistically, inhibiting BMI1 can reverse this ubiquitination and silencing effect, increasing CD8+ T cell infiltration and enhancing anti-tumor immune activity [175]. In multiple myeloma, transcriptional epigenetic regulation by m6A methylation can modulate an oncogenic transcription factor, ETV1 (E-twenty-six transformation-specific variant 1), and increase its expression [176]. Overexpression of ETV1 leads to polarization of TAMs, contributing to disease progression in this malignancy.

Taken together, this demonstrates how epigenetics can regulate macrophage-mediated immune evasion at multiple levels, impacting macrophage polarization, immune signaling and immunosuppressive capacity in blood cancers. Importantly, these studies highlight the therapeutic potential of targeting epigenetic pathways that reprogram macrophages and other immune cells to restore anti-tumor immunity in hematologic malignancies.

## 6. Targeting the Immune System with Epigenetic Modulation

**Figure 2:**
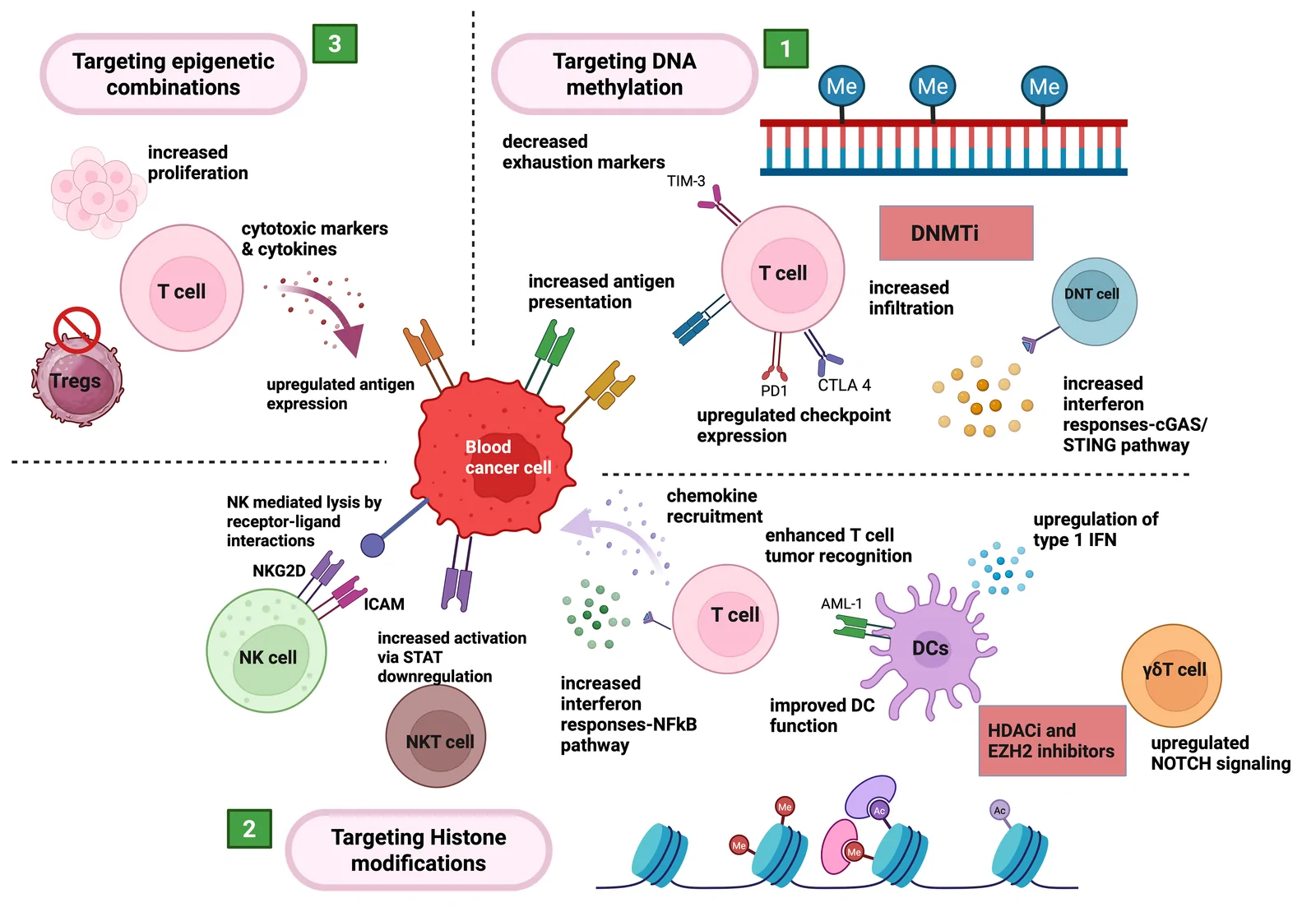
Using epigenetics to improve immune cell function in hematologic malignancies. Schematic illustrating how targeting distinct epigenetic mechanisms can modulate and enhance immune cell function in blood cancers. (1) Targeting DNA methylation with hypomethylating agents (azacytidine, decitabine) enhances T cell function by increasing T cell infiltration, reducing exhaustion marker expression (e.g., TIM-3), and promoting antigen presentation [183,190,199,224]. However, these agents can also upregulate inhibitory checkpoint molecules (e.g., PD-1, CTLA-4, PD-L1), which may contribute to immune escape [181]. In double-negative T (DNT) cells, DNMTis enhance interferon gene expression and activate the cGAS/STING pathway, thereby promoting anti-tumor immunity [189]. (2) Targeting histone modifications using HDACi and EZH2i can enhance NK cell-mediated cytotoxicity (via NKG2D, etc). These inhibitors also promote NKT cell activation through modulation of STAT signaling [220]. In T cells, histone-targeting therapies improve tumor recognition, enhance interferon response genes and NF-κB signaling, and promote chemokine production and immune cell recruitment [127,207,213,217]. In DCs, these agents induce type I interferon signaling and NOTCH signaling in γδ T cells [212,216]. (3) Combinatorial epigenetic therapies further potentiate anti-tumor immunity by promoting effector T cell proliferation, increasing tumor antigen expression, enhancing cytotoxic molecule and cytokine production, and suppressing regulatory T cell function, collectively improving anti-tumor immune responses [224,225,226,227]. Figure created in bioRender.com.

Given the contribution of epigenetic dysregulation to both tumor immune escape and immune cell dysfunction, epigenetic therapies may also directly improve immune cell function, in addition to their direct effect on tumor cells (Figure 2). This section focuses on targeting DNA methylation to modulate antigen presentation, inflammatory signaling, immune cell function and checkpoint expression, followed by immune modulatory effects of histone-targeted therapies.

## 7. Targeting DNA Methylation

### 7.1. Antigen Presentation and Expression

Beyond their cytotoxic effects, DNA methyltransferase inhibitors (DNMTis) such as azacytidine (Aza) and decitabine have shown immunomodulatory effects which are now actively being explored in hematologic malignancies. One major observation is that Aza can enhance the expression of cancer testis antigens (CTAs), which are usually restricted to sites that are immune-privileged [177,178]. This enhanced expression may improve leukemia recognition by T cells. In addition, Aza can increase the expression of MHC molecules, potentially enhancing antigen presentation in both solid cancers and hematologic malignancies [179,180].

However, the immunologic effects of DNMT inhibitors are context-dependent and should not be considered uniformly immune-activating. In addition to promoting antigen presentation and inflammatory signaling, Aza and decitabine can induce myelosuppression, alter lymphocyte proliferation, expand regulatory T cell populations in certain settings, and increase expression of inhibitory immune checkpoints including PD-1 and PD-L1 [116,181,182]. Consequently, the net immunologic effect of DNMT inhibition likely reflects a balance between enhanced tumor recognition and induction of compensatory immune-suppressive mechanisms. Furthermore, Aza and decitabine should not be considered fully interchangeable agents, as differences in pharmacology, RNA versus DNA incorporation, and treatment schedules may influence their biological and immune-modulatory effects.

Consistent with these findings, an ex vivo assay set up using peripheral blood samples from patients with AML demonstrated better T cell recognition of tumor after Aza-treatment, as measured by degranulation marker CD107a, indicating cytotoxic activity. This effect coincided with increased CTA expression [183]. However, while this correlation suggests enhanced immune recognition, this study did not directly establish if CTA expression was mechanistically responsible for this enhanced T cell cytotoxic activity. Similarly, studies using decitabine also show induction of CTAs including NY-ESO and MAGE that is usually associated with improved T cell recognition and function in hematologic malignancies [184,185,186].

### 7.2. Inflammatory Signaling and Immune Cell Function

DNMTis may activate interferon signaling pathways. Early studies in solid tumors showed that Aza can activate type I interferon pathway and that blocking this interferon-α/β signaling abrogated this effect [187]. This group also reported that transcripts of endogenous retroviruses were upregulated after DNMTi treatment, which activated this innate signaling pathway. Similar observations have also been implicated in AML [188,189], where Aza utilized the cGAS-STING pathway to enhance T cell-mediated killing of tumor [189]. Upon inhibiting this pathway using pharmacologic inhibition of STING, Aza lost this anti-tumor effect. Moreover, Aza treatment also led to an increased expression of type I interferons in this setting. Given the central role of interferon signaling in T cell activation and antigen presentation, this pathway represents an important mechanism through which Aza may modulate anti-tumor immunity.

Clinical studies also support these immunomodulatory findings. A phase III clinical trial examining Aza as a maintenance therapy for patients with AML that were in remission after chemotherapy found that Aza was associated with increased T cell infiltration and reduced expression of T cell exhaustion markers in the bone marrow, correlating with better overall survival [190]. This suggests Aza may help modulate T cell exhaustion states and needs to be further investigated. Additional clinical trials across hematologic malignancies also reported immune-modifying effects of DNMTis [191,192]. Interestingly, similar principles have emerged from studies in chronic viral infection and CAR-T cell models, where it is shown that a major cause of T cell exhaustion is *de novo* DNA methylation. As a result, genetic or pharmacologic inhibition of these DNA methylation programs restored T cell function [193,194], highlighting the therapeutic potential of targeting DNA methylation in this context as well.

In addition to T cell effects, Aza can also modulate NK cell function. In a clinical trial of patients with MDS, Aza treatment enhanced NK cell function, including an increase in KIRs, enhanced cytokine response and upregulated cytotoxic markers [195]. Other studies highlighted in this review support these findings as well.

### 7.3. Immune Checkpoint Regulation

In addition to enhancing immune cell function, hypomethylating agents also modulate expression of several inhibitory checkpoint markers. Peripheral blood collected from patients with AML/MDS treated with Aza showed promoter demethylation of *PDCD1,* which was associated with higher PD-1 expression on T cells. Interestingly, higher PD-1 methylation was found in patients that were non-responders to Aza therapy [116]. Another study demonstrated that exposure to hypomethylating agents led to an increased expression of PD-1, PD-L1, PD-L2 and CTLA-4 across multiple AML cell lines and patient samples [181], highlighting the complex interplay between epigenetic therapy and immune modulation. DNMTis may also reverse tumor-mediated NK cell evasion via checkpoint modulation. For example, AML blasts often downregulate ICAM, a ligand recognized by NK cells to escape immune surveillance. Treatment with decitabine restored ICAM expression, resulting in enhanced NK cell activation and their cytotoxicity against leukemia in vitro and in vivo [97]. Other studies support the role of decitabine in improving NK cell function in other hematologic cancers as well [196,197].

Despite these promising immune-modifying effects, clinical responses to DNMTis, especially Aza, remain low, and relapse rates remain high. Although resistance mechanisms are multifactorial, inhibitory checkpoint upregulation may contribute to therapy resistance [198]. In a syngeneic mouse model of AML, the authors demonstrated that Aza increased T cell infiltration but also induced a higher expression of inhibitory checkpoint markers [199], consistent with previous observations highlighted earlier in this section. This upregulation provides part of the rationale for combining Aza with checkpoint blockade therapy.

However, clinical studies evaluating these combinations have yielded limited success so far. Moreover, a recent clinical trial combining Aza with PD-1 blockade showed unfavorable clinical outcomes [200], suggesting this checkpoint upregulation alone is unlikely to fully predict therapy benefit from checkpoint blockade. Ongoing checkpoint blockade trials in AML are also based on correlative observations, and the precise mechanisms by which hypomethylating agents (HMAs) reshape the immune landscape are poorly understood. Therefore, it would be helpful to get a deeper understanding of how different DNMTis regulate immune cell function, exhaustion, and antigen presentation. This will be important for optimizing combination therapies involving checkpoint inhibitors, additional epigenetic modulators, or other immunotherapeutic approaches.

Another important unresolved question is the temporal relationship between HMA-induced immune remodeling and checkpoint blockade. Although HMAs increase expression of inhibitory checkpoint molecules including PD-1, PD-L1, and CTLA-4, this phenomenon may reflect an adaptive resistance response that emerges following enhanced immune activation rather than a purely immunosuppressive effect [116,181]. Enhanced immune activation due to exposure to HMAs may trigger compensatory upregulation of inhibitory checkpoint pathways that ultimately limit anti-tumor responses. Under this model, checkpoint inhibitors function not to reverse the primary effects of HMAs, but rather to prevent or overcome the adaptive resistance induced by epigenetic priming. Scheduling may be critical, with epigenetic therapy administered before or during checkpoint blockade creating a more favorable immune state than checkpoint inhibition alone. Although definitive clinical evidence supporting sequential versus concurrent administration remains unknown, optimization of treatment timing and immune kinetics will likely represent an important area of future investigation.

## 8. Targeting Histone Modifications

Aberrant histone modification is a hallmark of many cancers, including blood cancers [201,202]. HDACs are often overexpressed and are associated with poor clinical outcomes, where they partly work by recruiting oncogenes that can drive leukemia progression [203]. Similarly, dysregulated histone methylation contributes to aberrant gene expression across several malignancies [204]. These findings have led to the development of several therapies directly targeting histone-modifying enzymes, including histone deacetylase inhibitors (HDACis) and histone methyltransferase inhibitors [205]. Indeed, several HDACis including panobinostat, romidepsin, and vorinostat are already clinically approved for certain hematologic malignancies [206] including cutaneous T cell lymphoma, multiple myeloma and peripheral T cell lymphoma. Although these agents were initially developed to target tumor cells, emerging evidence suggests that they also exert immune-modulating effects which may enhance anti-tumor immunity.

Inhibiting HDACs may enhance antigen presentation and lead to T cell priming. In AML, an HDACi, I13, increased expression of HLA class I and class II molecules [207] in cell lines, suggesting an immune-therapeutic potential. Similarly, inhibition of HDAC3 restored MHC class II expression in CREBBP-mutated lymphomas using multiple experimental systems [127]. This also partially restored impaired interferon pathways linked with immune evasion. HDACis also improve the function of antigen-presenting cells. In cancers including AML and CML, leukemic cells also possess the capacity to be reprogrammed to dendritic cell (DC)-like populations [208,209]. Because DCs are professional antigen presentation cells required for T cell priming, restoring their effects is another promising strategy to overcome immune evasion. DCs generated from AML precursors often lack potency and display impaired maturation and antigen presentation [210]. Treatment with HDACis enhanced both phenotypic and functional characteristics of leukemic DCs, including improvement in the presentation of AML-1 fusion protein, a tumor antigen which then enhances T cell-mediated cytotoxicity [211]. In another study, HDAC inhibition also increased type I interferon production by plasmacytoid DCs, improving therapeutic outcomes in AML samples treated with panobinostat [212].

Histone-modifying therapies may also regulate T cell functional states which is crucial for anti-tumor immunity. In an adoptive transfer murine model of CLL, HDAC6 inhibition led to a decrease in T cell exhaustion markers while increasing MHC class I and class II expression, supporting a role of HDAC inhibition in modulating anti-tumor immune responses in CLL [213]. HDACis also modulate γδT cells. These unconventional T cells possess anti-tumor immunity and are increasingly being explored for immune therapy potential [214,215]. Treatment in AML models using a pan HDACi upregulated NOTCH signaling pathway in γδT cells, boosting an anti-tumor cytotoxic effect [216]. Interestingly, this change was accompanied by reduced proliferation in these T cells, suggesting the importance of optimizing HDACi dosing and timing when integrating these agents into enhance immunotherapy.

In follicular lymphoma, inhibiting a histone methyltransferase EZH2 upregulated the expression of CCL17, a chemokine that recruits T cells. This promoted T cell infiltration and supported a potential role in restoring anti-lymphoma immunity [217]. This correlation between EZH2 and CCL17 was only observed in a subset of patients, suggesting inter-patient heterogeneity in response to epigenetic therapies.

HDACis also modify NK and NKT cell-mediated immune responses [216,218,219]. In mantle cell lymphoma (MCL) cell lines, treatment with a pan HDACi, Trichostatin A (TSA), enhanced MHC class II antigen presentation to NKT cells, facilitating their activation [220]. Mechanistically, HDAC inhibition downregulated the STAT signaling pathway, reducing the secretion of immune-suppressive cytokines associated with immune escape [220]. Similarly, in multiple myeloma cell lines, treatment with an HDACi, valproic acid, increased the expression of NKG2D ligand on myeloma cells, possibly mediated by activation of phosphorylated extracellular signal-regulated kinase (pERK) signaling [221]. This in turn enhanced NK degranulation and cytotoxic effects, promoting effective tumor killing.

Overall, these studies highlight that targeting histone modifications may shape anti-tumor immunity in hematologic malignancies. These findings also support the rationale for combination strategies involving multiple epigenetic agents and/or immunotherapy to achieve better tumor outcomes. Some of these therapies are discussed below.

## 9. Epigenetic Combination Therapy

As mentioned, monotherapy with epigenetic modifiers, including DNA methyltransferase inhibitors (DNMTis) and histone deacetylase inhibitors (HDACis), has demonstrated clinical benefit in several cancers [222,223]. However, their efficacy remains limited due to therapy resistance, toxicity, off-target effects, incomplete immune activation, etc. Despite the challenges, combining different classes of epigenetic therapies has emerged as a promising strategy to overcome some of these limitations and enhance anti-tumor immune effects.

### 9.1. Combined HDAC and DNMT Regulated Antigen Expression

Recognition of tumor-associated antigens is pivotal for generating a robust response against the tumor [228,229]. Members of the CTA family are an attractive immunotherapy and vaccine target due to their high immunogenicity [230,231,232]. Combination treatment with DNMTi and HDACi enhanced expression of MAGE antigens in AML and MDS, a CTA family antigen. This was accompanied by an increase in cytotoxic T cell responses against these antigens and associated with improved therapeutic response in patients [225]. However, in this study Aza was the primary driver of this induction, and the contribution of individual therapy was not established in this study. Another study in AML reported similar regulation of PRAME expression [226]. Consistent findings have been reported in multiple myeloma [224], where combining Aza with HDACi MGCD0103 increased expression of the MAGE-A3 antigen in multiple myeloma cell lines, leading to enhanced T cell recognition. Similar to the other study, addition of HDACi to Aza enhanced this antigen expression and was not seen with MGCD0103 alone. Therefore, even though DNMT inhibition may be the primary driver of increased antigen expression, adding HDACi may further enhance T cell recognition via antigen expression.

### 9.2. Combined HDAC and DNMT Regulated Immune Suppression

In addition to increasing tumor antigen expression, epigenetic drug combinations also modulate immunosuppressive populations within the tumor microenvironment. Tregs are major mediators of immunosuppression in cancers and are often correlated with poor prognosis and immune evasion [9,17,233]. In AML, a highly suppressive Treg population that expresses TNF receptor 2 (TNFR2+) is enriched within the bone marrow. Treatment with Aza and panobinostat in patient samples significantly reduced these highly functional Tregs and increased the expression of cytokines important for T cell function, supporting the immune-modifying potential of this combination [227]. Aza alone has previously been shown to induce Tregs through demethylation of the FoxP3 promotor [182], suggesting this observed reduction was mainly driven by panobinostat. Therefore, the precise contribution of Aza in modulating these Tregs is incompletely understood and warrants further investigation. Combination epigenetic therapies also impact other immune cell populations involved in anti-tumor immunity. In an immunocompetent murine model of multiple myeloma, combination treatment using decitabine and quisinostat (HDACi) promoted DC maturation, reduced expression of inhibitory immune checkpoint markers on the tumor and decreased T cell exhaustion markers, although decitabine was the main driver of these effects [234].

Collectively, these studies highlight that epigenetic combinations may integrate distinct effects on tumor immunogenicity and immune responses in hematologic malignancies, although the contribution of each agent varies across disease settings. These findings provide a rationale for combining these agents with immune therapy, which is discussed below.

## 10. Combining Epigenetic and Immune Therapies

Given the growing evidence that epigenetic dysregulation may play a critical role in modulating anti-tumor immunity, there is rationale for combining epigenetic therapies with immunotherapeutic approaches in hematologic malignancies. Several preclinical and clinical studies have now begun exploring these strategies to enhance immune function and overcome therapy resistance.

## 11. Combined Checkpoint Inhibition and Epigenetic Drug Therapy

A highly active area of investigation is the combination of checkpoint blockade with epigenetic therapies in hematologic malignancies. As previously discussed, the rationale for these combinations stems from observations that HMAs upregulate checkpoint molecule expression including PD-1, PD-L1 and CTLA-4 in AML and other hematologic malignancies, providing an opportunity for combined blockade strategies. Based on these observations, several studies in AML and MDS evaluated combining hypomethylating agents with immune checkpoint inhibitors including pembrolizumab, durvalumab and ipilimumab [198]. Even though some of these combinations demonstrated therapeutic potential, as mentioned earlier, the responses were not durable [200,235,236]. Although several trials are still evaluating targeting these checkpoints, other immune checkpoints have also gained interest in these settings. In patients with high-risk MDS and CMML, clinical trials combining TIM-3 inhibitor sabatolimab with standard-of-care hypomethylating agents showed promising safety, positioning TIM-3 as a therapeutic agent for these malignancies [237,238]. Other studies have shown promise of targeting additional immune checkpoints with HMAs in hematologic malignancies as well [239,240]. Therefore, combining hypomethylating agents with checkpoint blockade therapies still remain an active area of clinical investigation, although identifying the relevant inhibitory checkpoint pathways in different disease settings will be crucial for showing clinical benefit.

HDACis are also being investigated in combination with checkpoint blockade in malignancies including prostate and renal cancers and melanoma [241,242]. PD-1 blockade is also clinically used in a subset of patients with Hodkin’s lymphoma, but not all patients with lymphomas respond. A study using a lymphoma mouse model showed that combining an HDACi, OKI-179, with PD-1 blockade increased MHC class II expression, activated T cells and hence improved tumor control [243]. Similarly, another study using mouse models of NKT cell lymphoma found that HDACis when combined with PD-1 blockade enhanced tumor control and increased T cell-specific cytotoxic cytokine response [244]. These studies suggest HDACis combined with PD-1 blockade can be beneficial, with some studies suggesting synergistic anti-tumor effects accompanied by improved T cell responses.

In addition to DNA methylation and histone-targeting agents, other epigenetic regulators have also been implicated in immune evasion and immune therapy resistance. Bromodomain and extra terminal domain (BET) proteins are epigenetic readers that play a critical role in several processes including chromatin accessibility and regulating transcription via histone acetylation [245]. Among these, a BET family member BRD4 has emerged as a critical regulator of the key oncogenic pathway including MYC, therefore contributing to tumor progression [246,247]. These proteins have been shown to promote tumor growth and survival in several cancers including melanoma, leukemias, myelomas, and breast cancers via multiple mechanisms which also include NF-κB and PI3K/AKT pathways [248,249]. As a result, BET inhibitors (BETis) have gained interest as therapeutic agents in solid tumors and hematologic malignancies [250,251]. In hematologic malignancies including lymphomas, AML and ALL, BETis have shown promising anti-tumor activity in preclinical studies and are being evaluated in clinical trials [252]. Beyond their impact on tumor cells, they can also modulate immune responses, providing a rationale for combining them with immunotherapies including checkpoint blockade [248,253,254,255]. For example, BETis can downregulate PD-L1 in lymphomas by disrupting the transcriptional regulation of CD274 mediated by BRD4 [256]. Additionally, combination treatment with BETis and PD-1 blockade further enhanced anti-tumor activity in preclinical models. Supporting these findings, a recent study from our lab showed that combining BET inhibition with PD-1 blockade in AML reversed T cell exhaustion and increased the chromatin accessibility of TCF7, the transcription factor associated with stem-like T cell states and durable anti-tumor responses [257].

### Toxicity Considerations of Epigenetic–Immune Combination Therapies

Although combining epigenetic therapies with immunotherapy has shown promise in preclinical studies, treatment-related toxicity remains a major challenge to clinical implementation. Across hematologic malignancies, the most common adverse events reported with these combinations are hematologic toxicities, including neutropenia, thrombocytopenia, anemia and infection-related complications. Because many of these studies are conducted in patients with advanced leukemia, lymphoma or myelodysplastic syndromes who already have baseline cytopenias, distinguishing treatment-related toxicity from disease-related hematologic dysfunction remains difficult.

Clinical studies evaluating HMAs with immune checkpoint blockade have generally reported manageable safety profiles but have not consistently demonstrated improved efficacy. For example, randomized studies of Aza combined with durvalumab in AML and high-risk MDS did not identify new safety signals, although grade 3–4 hematologic adverse events remained frequent and, in some settings, occurred at higher rates than with Aza alone. Immune-related adverse events including hepatitis, pneumonitis, endocrinopathies and dermatologic toxicities also remain important considerations when checkpoint inhibitors are incorporated into epigenetic regimens. While most studies report manageable rates of these toxicities, severe immune-mediated events may necessitate treatment interruption or discontinuation [236,258].

Similar observations have been reported with HDACi-based combinations. The combination with anti-PD1 was assessed in a phase Ib study in AML and MDS. Toxicity was observed but generally manageable, predominantly driven by myelosuppression. Grade 3–4 thrombocytopenia, neutropenia, and anemia were the most common adverse events, while infections, fatigue, and diarrhea were the most frequently reported non-hematologic toxicities. Immune-related adverse events, including pneumonitis, were observed in a subset of patients, and treatment interruptions or dose modifications were commonly required [259]. These findings indicate that HDACi–immunotherapy combinations are feasible but require careful monitoring for hematologic and immune-mediated toxicities.

Strategies to improve the therapeutic index of epigenetic–immune combinations will likely require optimization of dose and scheduling, including evaluation of sequential rather than concurrent administration, development of signatures that identify patients most likely to benefit, and improved understanding of the mechanisms driving both efficacy and toxicity. Such approaches may allow selective enhancement of anti-tumor immunity while minimizing treatment-limiting adverse events.

Despite compelling preclinical evidence, the clinical activity of HMA–checkpoint inhibitor combinations in AML and MDS has been variable. Several factors may contribute to this translational gap, including substantial disease heterogeneity at both the clonal and patient level [260,261], differences in the immune microenvironment [262], and the presence of profound baseline T cell dysfunction that may not be fully reversible with checkpoint blockade alone. Clinical outcomes may also be influenced by prior therapies, particularly previous HMA exposure, which has been associated with lower response rates in some studies [263]. Furthermore, treatment-related cytopenias, infections, and immune-related toxicities can limit therapy delivery and sustained immune activation. The optimal dosing, timing, and sequencing of HMAs and checkpoint inhibitors remain unknown, and the lack of validated predictive biomarkers continues to hinder identification of patients most likely to benefit from these approaches. Recently, a signature related to the frequency of tumor-reactive T cells has been shown to predict the rare responders in PD-1 + Aza trials, but this signature was based on a small subset of patients [264]. Collectively, these challenges highlight the need for better markers for patient selection and more rationally designed combination strategies.

## 12. Epigenetic Modulation in Adoptive Cell Therapy

CAR-T cell therapies are FDA-approved for several B cell malignancies and are another promising platform for combination with epigenetic therapies. Another area of interest involves combining epigenetic therapies with adoptive T cell-based therapies. In mouse models of AML and MM, investigators evaluated combining Aza with a SUMOlytion inhibitor, TAK981, which was shown to improve tumor cell killing [265], together with a TCR engineered T cell therapy. The triple combination with TCR therapy, Aza and TAK981 led to improved T cell effector function, cytokine production and proliferation and enhanced tumor recognition [266]. While these results are promising, mechanisms underlying these effects are incompletely understood. An inherent challenge highlighted by the authors here is technical difficulties associated with isolating immune cells from mouse bone marrow due to their low abundance in those tissue sites, underscoring a broader challenge in studying immune function in hematologic malignancies. Future studies should incorporate immune cell enrichment strategies and perform special multi-omics technologies to address this challenge.

Aza can upregulate antigen expression on tumor cells and thus may help overcome some of the key challenges associated with CAR-T cells, including antigen heterogeneity and immune escape. A CD123 CAR-T cell alone demonstrated limited efficacy but, when combined with Aza, increased AML tumor killing was observed due to upregulation of CD123 on tumor cells, which also improved T cell function [267]. This study further suggested that Aza may regulate some of these effects by modulating the phosphorylation of proteins involved in T cell activation and signaling. Similarly, another study demonstrated that in AML combining Aza with CD70 CAR-T cells enhanced anti-leukemic efficacy. Aza increased CD70 antigen density by promoter hypomethylation, and that combination therapy led to enhanced anti-tumor effects in murine models [268]. Similar results have been observed with decitabine as well in leukemia and lymphoma [269,270,271]. This suggests hypomethylating agents can enhance CAR-T cell outcomes across multiple antigen targets in hematologic malignancies.

Studies have also focused on combining histone-modifying therapies with CAR-T cells. Combining EZH2 inhibition with CD19 CAR-T cells can augment its therapeutic activity, accompanied by increasing tumor killing andcapacity, enhancing expansion of CAR T cells in lymphoma mouse models [272]. The authors report that the inhibition needs to occur within the tumor to have a therapeutic effect, suggesting the observed effects were mediated by tumor-intrinsic mechanisms rather than direct effect on CAR-T cell function. Another study extended this idea by showing that EZH2 inhibition can also enhance CAR-T cell and other T cell engineered therapies by effects on both tumor and T cells, including reduced T cell exhaustion and memory formation [74]. This study was done in R/R NHL mouse models and is being evaluated clinically. Similarly, some HDACis can improve function of CAR-T cells in preclinical models by enhancing memory formation and limiting exhaustion via activation of the canonical Wnt/β-catenin pathway, indicating a T cell-intrinsic effect [273]. Together these findings suggest that the impact of epigenetic inhibition can be highly context-dependent and varies with disease setting, timing of exposure and the cell compartment it targets.

Gene modifications of epigenetic regulators in CAR-T cells also modulate their effects. TET2 is a critical DNA demethylation enzyme and is extensively studied in hematologic malignancies and can also regulate T cell function and exhaustion [141,274,275,276]. Carl June’s lab showed that in CLL, loss of TET2 can be beneficial for CAR-T cell function [277]. They found that a patient that received CD19 CAR-T cell therapy achieved complete remission, and further analysis showed that 94% of the CAR-T cells were derived from a single clone that had an alteration of TET2. Notably, the patient also carried a preexisting mutation in the second TET2 allele. This biallelic mutation shifted the T cells towards a central memory phenotype and improved their persistence and was associated with improved therapeutic outcomes. Studies from the same group and others also extended these findings in other CAR-T settings, where modulation of TET2 restored anti-tumor efficacy [274]. TET2 deletion led to decrease in exhaustion, and increase in proliferation, memory potential and anti-tumor efficacy of CAR-T cells. Even downregulation of TET2 in CAR-T cells can improve tumor control [278,279]. However, a study also reported that biallelic TET2 loss in some settings led to aberrant clonal expansion of CAR-T cells [280], suggesting that we need to optimize the degree and duration of this modulation. DNMT3A, as mentioned earlier, is targeted by hypomethylating agents and plays a significant role in regulating T cell function and exhaustion as well. Multiple studies in both chronic viral infections and cancers showed that knocking out DNMT3A can reinvigorate T cell responses and reduce dysfunction associated with exhaustion [193]. Consistent with these findings, deletion of DNMT3A in CD19 CAR-T cells enhanced stemness and improved function [194]. Similarly, another study showed that deletion of a histone methyltransferase gene, *SUV39H1*, in CAR-T cells also enhanced their persistence and tumor control in a mouse model of B-ALL [281].

Beyond T cell-directed therapies, epigenetic therapies in combination with other immune cell types are also being investigated. For example, NK cell therapies in combination with Aza and venetoclax are currently being evaluated in a phase I clinical trial in AML. Also, in multiple myeloma, combining an HDACi, entinostat, with CAR-NK cells improved the function of NK cells and potentiated their anti-tumor effects.

An extensive list of ongoing clinical trials evaluating epigenetic agents in combination with checkpoint blockade and other immune therapies is provided in Table 1. However, it is critical to acknowledge that the benefit observed in preclinical studies has not yet fully translated to clinical settings. Briefly, the studies reported here show variable outcomes with epigenetic–immune combination therapies. Responses tend to be better in patients that were newly diagnosed or previously untreated compared to relapsed or refractory patients or those who failed prior therapies, although differences across studies and patient populations limit direct comparisons. Importantly, many of these trials were small, early-phase, and single-arm, making it challenging to determine if the immune-targeting agent provided benefit beyond the epigenetic therapy alone. In the relatively few studies that used monotherapy with epigenetic treatment, the addition of immune therapy did not improve response or survival consistently and was sometimes associated with higher toxicity. Adverse effects also varied across studies but commonly included infections and hematologic complications, as well as fatigue, anemia and nausea, with some studies reporting higher adverse events than others. Some studies were terminated due to strategic decisions made by sponsors, and were not based on toxicity, safety concerns or treatment response. Some studies remain in unknown status despite subsequent publication of clinical findings. Overall, these studies suggest that although epigenetic–immune combination can demonstrate clinical activity in some settings, their added therapeutic advantage remains limited, especially due to lack of comparison arms, limited benefits in certain patient populations including relapsed/refractory settings and variable toxicity. In some studies, limited immune-cell expansion or small patient numbers further restrict the interpretation of clinical activity.

Altogether, these preclinical and clinical studies highlight the growing potential and challenges of combining epigenetic therapies with immune therapy to enhance anti-tumor immune responses in blood cancers. However, mechanistic evidence remains variable across studies and benefit has not yet consistently translated to improve patient outcomes. A major limitation is that the individual contribution of each therapy is not often fully dissected, or a causal relationship between the observed epigenetic therapy and immune modulation is not always established. Prior treatment exposure, disease setting or patients’ immune landscape may also influence treatment response. Further studies are therefore needed to define the mechanistic pathways, optimize these strategies and identify patients that will most benefit from these combination strategies.

## 13. Conclusions and Future Directions

An overarching challenge in studying immune evasion in hematologic malignancies is the lack of suitable preclinical models that truly recapitulate tumor–immune interactions. Many studies rely on xenograft mouse models, which lack an intact immune system, therefore limiting the ability to study the complex immune dynamic within the tumor microenvironment. The limited availability of syngeneic leukemia models and murine-derived AML fails to recapitulate the large genetic diversity in blood cancers and remains a major hurdle for mechanistic studies and testing novel combinations. Many preclinical studies combining epigenetic therapies with T cells utilize healthy donor-derived T cells which might not accurately model the dysfunctional or exhausted phenotype typically observed in cancer patients. Developing models utilizing patient-derived or tumor-associated T cells will therefore be important for translating these findings in patients.

Despite these challenges, targeting epigenetic mechanisms of immune evasion in hematologic cancers is a promising area of investigation. The translational relevance of this field is highlighted by the success of epigenetic therapies in several blood cancers such as AML, multiple myeloma and certain lymphomas. However, therapy resistance is a major barrier to success of these therapies, emphasizing the need to better understand the mechanisms underlying both therapy response and relapse. As highlighted in this review, epigenetic dysregulation can impact several aspects of anti-tumor immunity including antigen presentation, immune checkpoint expression, cytokine production, immune cell function, states and exhaustion across multiple immune cell types.

Epigenetic therapies can enhance responsiveness to checkpoint blockade and other immune therapies. However, it is important to understand that many current studies are largely descriptive or correlative, and mechanistic insights on how these combinations modulate anti-tumor immunity are still limited. Understanding these pathways are critical in optimizing these combinational therapies for patients.

Finally, patient heterogeneity adds another layer of complexity to understanding epigenetic regulation and immune modulation in blood cancers. Advanced approaches including single-cell RNA sequencing, chromatin profiling, and multi-omics techniques will be essential to understand these complex interactions and develop relevant therapies. Continued mechanistic and translational investigation of epigenetic regulation of anti-tumor responses can improve immune therapy outcomes in patients with hematologic malignancies.

## Figures and Tables

**Table 1:** Clinical trials investigating epigenetic–immune therapy combinations in hematologic malignancies (obtained from ClinicalTrials.gov, accessed on 7 September 2026).

Combination Type	Drug Combination	Cancer Type	Patient Population	Phase	Status	NCT Identifier	URL to Clinical Trial
HMA + ICB	Pembrolizumab + azacytidine	AML	R/R, ND	II	Completed	NCT02845297	https://clinicaltrials.gov/study/NCT02845297
HMA + ICB	Pembrolizumab + azacytidine	MDS	Intermediate/high risk	II	Unknown, previously active, not recruiting	NCT03094637	https://clinicaltrials.gov/study/NCT03094637
HMA + ICB + Ven	Azacytidine + nivolumab + ipilimumab + venetoclax (in different combinations/arms)	AML	R/R, ND	II	Completed	NCT02397720	https://clinicaltrials.gov/study/NCT02397720
HMA + ICB	Pembrolizumab + decitabine	AML	R/R	I/II	Completed	NCT02996474	https://clinicaltrials.gov/study/NCT02996474
HMA + ICB	Sabatolimab + azacytidine	AML/secondary AML	CR with MRD + post-aHSCT	Ib/II	Terminated	NCT04623216	https://clinicaltrials.gov/study/NCT04623216
HMA + ICB	Azacytidine + durvalumab	MDS/AML	High-risk MDS/ND AML	II	Completed	NCT02775903	https://clinicaltrials.gov/study/NCT02775903
HMA + CD47	Azacytidine + magrolimab	MDS/AML	High-risk AML/MDS	I	Withdrawn	NCT05823480	https://clinicaltrials.gov/study/NCT05823480
HMA + CD70	Cusatuzumab + azacytidine	AML/MDS	High-risk MDS/ND AML	I/II	Completed	NCT03030612	https://clinicaltrials.gov/study/NCT03030612
HMA + HDACi	Azacytidine + romidepsin (vs. investigator’s choice)	PTCL	R/R	II	Active, recruiting	NCT04747236	https://clinicaltrials.gov/study/NCT04747236
HDACi + ICB	Pembrolizumab + entinostat	lymphomas	R/R	II	Active, not recruiting	NCT03179930	https://clinicaltrials.gov/study/NCT03179930
HDACi + ICB	Pembrolizumab + vorinostat	DLBCL/FCL/HL	R/R	I	Active, not recruiting	NCT03150329	https://clinicaltrials.gov/study/NCT03150329
HDACi + ICB	Pembrolizumab + romidepsin	PTCL	R/R	I/II	Active, not recruiting	NCT03278782	https://clinicaltrials.gov/study/NCT03278782
HDACi + HMA	Chidamine + decitabine	NHL	R/R (after CAR-T infusion)	I/II	Unknown, previously recruiting	NCT04337606	https://clinicaltrials.gov/study/NCT04337606
HDAC + HMA + ICB	Decitabine + camrelizumab + chidamine	CHL	R/R	II	Unknown, previously recruiting	NCT04233294	https://clinicaltrials.gov/study/NCT04233294
HMA + TAA	NY-ESO-1, MAGEA4, PRAME, survivin and SSX + azacytidine	HL/NHL	Active/relapsed	I	Active, not recruiting	NCT01333046	https://clinicaltrials.gov/study/NCT01333046
HMTi + ICB + MAb	Tazemetostat + atezolizumab + obinutuzumab	FL/DLBCL	R/R	Ib	Completed	NCT02220842	https://clinicaltrials.gov/study/NCT02220842
HDACi + ICB	Entinostat + PD1	MDS	DNMTi failure	I	Completed	NCT02936752	https://clinicaltrials.gov/study/NCT02936752
HDACi + HMA	Azacytidine + MS-275	MDS/CML/AML		I	Completed	NCT00101179	https://clinicaltrials.gov/study/NCT00101179
HMA + T cells	Azacytidine + DLI	AML	High-risk SCT	II	Not yet recruiting	NCT06754540	https://clinicaltrials.gov/study/NCT06754540
HMA + NK engager + Ven	Azacytidine + NK-targeting CD123 + Ven	AML	ND, unfit for chemo	I/II	Terminated	NCT06508489	https://clinicaltrials.gov/study/NCT06508489
HMA + CAR-T + Ven	Azacytidine + CD19CD22 CAR-T + Ven	B-ALL	ND, high risk	II	Unknown, previously recruiting	NCT06078306	https://clinicaltrials.gov/study/NCT06078306
HMA + NK cells + Ven	Azacytidine + allogeneic NK cells + Ven	AML	Unfit for chemo or allo SCT	Ib	Active, recruiting	NCT05834244	https://clinicaltrials.gov/study/NCT05834244
HMA + CD70Mab + Ven	Azacytidine + cusatuzumab + Ven	AML	De novo or secondary, unfit for chemo	Ib	Active, not recruiting	NCT04150887	https://clinicaltrials.gov/study/NCT04150887
HMA + macrophage + Ven	Azacytidine + bexmarilimab + Ven	MDS/CMML/AML	Int/HR MDS, 10–19% blasts in CMML or HMA failure, ND/R/R AML	I/II	Active, not recruiting	NCT05428969	https://clinicaltrials.gov/study/NCT05428969
HDACi + ICB	Chidamide + sintilimab	PTCL	R/R	II	Active, recruiting	NCT04512534	https://clinicaltrials.gov/study/NCT04512534
HDACi + HMA + ICB	Chidamide + decitabine + anti-PD-1 vs. standard	cHL	Refused/ineligible for transplant	II	Active, recruiting	NCT06393361	https://clinicaltrials.gov/study/NCT06393361
HDACi + HMA + NK cells	Vorinostat + decitabine + CD3/CD19-enriched donor NK cells + IL-2	MDS	High risk	II	Completed	NCT01593670	https://clinicaltrials.gov/study/NCT01593670
HMTi + CAR-T cells	Tazemetostat + CAR-T cells	DLBCL, FL, MCL (previously treated)	Confirmed disease, eligible for CAR-T therapy	I	Active, not recruiting	NCT05934838	https://clinicaltrials.gov/study/NCT05934838

List of clinical trials evaluating epigenetic–immune combinations. AML = acute myeloid leukemia, MDS = myelodysplastic syndromes, HMA = hypomethylating agents, ICB = immune checkpoint blockade, R/R = relapsed/refractory, ND = newly diagnosed, MRD = minimal residual disease, SCT = stem cell transplant, CR = complete response, Ven = venetoclax, PTCL = peripheral T cell lymphoma, DLI = donor lymphocyte infusion, DLBCL = diffuse large B cell lymphoma, FL = follicular lymphoma, HL = Hodgkin lymphoma, NHL = non-Hodgkin lymphoma, CHL = classical Hodgkin’s lymphoma, TAA = tumor-associated antigens, MCL = mantle cell lymphoma, CMML = chronic myelomonocytic leukemia.

## Data Availability

No new data were created or analyzed in this study. Data sharing is not applicable to this article.
